# Interplant signal transduction between dodder (*Cuscuta*) and their hosts

**DOI:** 10.1093/pcp/pcaf117

**Published:** 2025-10-19

**Authors:** Jingxiong Zhang, Guojing Shen, Songkui Cui, Wentao Wang, Jianqiang Wu

**Affiliations:** Department of Economic Plants and Biotechnology, Yunnan Key Laboratory for Wild Plant Resources, Kunming Institute of Botany, Chinese Academy of Sciences, Lanhei, Panlong, Kunming 650201, China; Department of Economic Plants and Biotechnology, Yunnan Key Laboratory for Wild Plant Resources, Kunming Institute of Botany, Chinese Academy of Sciences, Lanhei, Panlong, Kunming 650201, China; State Key Laboratory of Plant Diversity and Specialty Crops, Xiangshan, Haidian, Beijing 100093, China; Department of Economic Plants and Biotechnology, Yunnan Key Laboratory for Wild Plant Resources, Kunming Institute of Botany, Chinese Academy of Sciences, Lanhei, Panlong, Kunming 650201, China; State Key Laboratory of Plant Diversity and Specialty Crops, Xiangshan, Haidian, Beijing 100093, China; Department of Economic Plants and Biotechnology, Yunnan Key Laboratory for Wild Plant Resources, Kunming Institute of Botany, Chinese Academy of Sciences, Lanhei, Panlong, Kunming 650201, China; Department of Economic Plants and Biotechnology, Yunnan Key Laboratory for Wild Plant Resources, Kunming Institute of Botany, Chinese Academy of Sciences, Lanhei, Panlong, Kunming 650201, China; State Key Laboratory of Plant Diversity and Specialty Crops, Xiangshan, Haidian, Beijing 100093, China; CAS Center for Excellence in Biotic Interactions, University of Chinese Academy of Sciences, Yuquan, Shijingshan, Beijing 100049, China

**Keywords:** systemic signals, mobile signals, parasitic plant, host plant–parasitic plant interactions

## Abstract

Parasitic plants partly or completely depend on their host plants for growth and development. Through haustoria, parasitic plants extract water and nutrients from their hosts. However, there is also evidence that various biomolecules, including systemic signals, mRNAs, small RNAs (sRNAs), and proteins, are transferred between parasites and host plants and even among host plants commonly connected by a parasite. Many of these biomolecules transferred among plants may confer specific functions to recipient plants, altering their physiology and/or ecology. In this review, we summarize the current understanding of interplant systemic signaling between hosts and dodder (*Cuscuta*, Convolvulaceae), including the physiological and ecological functions of interplant systemic signals and the mechanisms underlying these functions. Next, the transfer of mRNAs, sRNAs, and proteins between hosts and dodder plants is reviewed, and the functions of these macromolecules are discussed.

## Introduction

**Figure 1:**
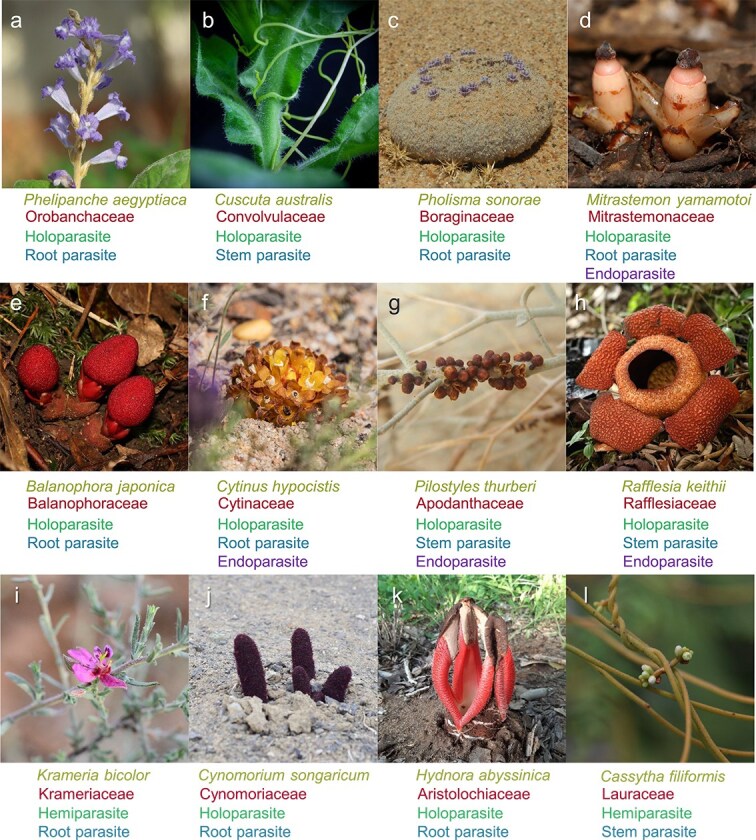
Representative photos of parasitic plants. Photos of parasitic plants from 12 angiosperm lineages. Photos by Jingxiong Zhang (a and b), Xingchen Jiang (c and g), Runxian Yu (d, e, f, k, h, and l), Jianong Li (f), Shuai Liao (i), Ziyuan Zhang (j), and Neng Wei (k).

Between 4000 and 5000 angiosperm plant species are parasites (Fig. 1), and are believed to have evolved independently from 12 to 13 ancestors (Westwood et al. 2010). These parasitic plants use a specialized organ, the haustorium, to attach to and penetrate the roots or shoots of host plants, extracting water and nutrients to support their own growth and development (Yoshida et al. 2016). Some parasitic plants, such as witchweeds (*Striga*, Orobanchaceae), broomrapes (*Orobanche* and *Phelipanche*, Orobanchaceae), dodders (*Cuscuta*, Convolvulaceae), and mistletoes (*Phoradendron*, *Viscum*, and *Arceuthobium*, Viscaceae), pose significant threats to agriculture and forestry. Consistent with their parasitic lifestyles, many parasitic plants, particularly the holoparasites, exhibit remarkable morphology and physiology, including loss of photosynthetic activity and being root- and leafless. Genome sequencing has indicated large-scale gene loss events in the genomes of several parasitic plant species and has revealed the genetic basis of the specific evolution of parasitic plants (Sun et al. 2018, Vogel et al. 2018, Cai et al. 2021, Xu et al. 2022, Chen et al. 2023). Studies on the unique physiology, ecology, and evolutionary histories of parasitic plants have already greatly enriched our understanding of this interesting group of plants and have started to provide novel strategies for the control of parasitic plants in agriculture and forestry.

Dodders are the only parasitic genus of Convolvulaceae, and these parasites are widely distributed in many parts of the world. Dodders are leafless and rootless. Their stems twine around host stems and branches, and along their stems many haustoria are formed, which penetrate host stems or branches to draw water and nutrients. Dodders have a wide range of host plants, including many crops, shrubs, and trees (Dawson et al. 1994), and they are among the controlled parasitic weeds in agriculture and forestry.

**Figure 2:**
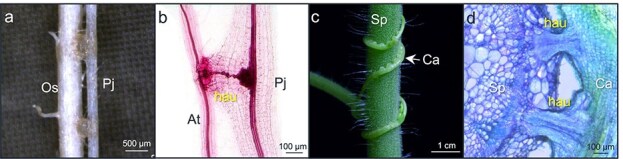
Haustorial connections between host plants and *Phtheirospermum japonicum* and between host plant and *Cuscuta australis*. (a) Photo of *Phtheirospermum japonicum* parasitizing a rice (*Oryza sativa*) root. (b) Safranin-O staining of the xylem bridge connection between an Arabidopsis root and *P. japonicum*. (c and d) Photo of *Cuscuta australis* parasitizing *Solanum pennellii* (c) and cross-sectional view of a *C. australis* haustorial structure stained with toluidine blue (d). Hau, haustorium; Os, *Oryza sativa*; At, *Arabidopsis thaliana*; Pj, *Phtheirospermum japonicum*; Sp, *Solanum pennellii*; Ca, *Cuscuta australis*.

Similar to the grafting, which relies on the formation of vascular connections between stock and scion (Feng et al. 2024), parasitic plants, including dodders, also establish vascular connections with their host plants, though they do so via haustoria. The xylem and phloem of many holoparasites are believed to be fused with the xylem and phloem, respectively, of their host plants, while most hemiparasites only fuse their xylem with that of hosts (Fig. 2) (Yoshida et al. 2016). Increasing lines of evidence, especially from the research on dodders, have revealed that there is interplant transfer of various biomolecules, including signaling molecules, mRNAs, small RNAs, and proteins, between parasitic plants and their host plants (Shen et al. 2023). These foreign biomolecules may confer signaling functions and affect the recipient plants’ physiology and/or ecological interactions with the environment. In this review, we summarize the inter-plant signal transduction and exchanges of macromolecules between dodder and their hosts, as well as between other parasitic plants and their hosts.

## Interplant Systemic Signaling between Dodder and Host Plants

Plants coordinate their growth, development, and response to environmental stresses across cells, tissues, and organs through systemic/mobile signals that control the corresponding physiological changes. Although the precise identities of most systemic signals remain elusive, they are believed to travel through the plant vascular system, in particular, the phloem. In host-parasitic plant systems, haustoria connect the vascular systems of hosts and parasites, facilitating the transfer of systemic signals that induce responses in the recipient plants.

### Herbivory-induced systemic signaling

Over 50 years ago, Green and Ryan (1972) demonstrated that wounding the leaves of tomato (*Solanum lycopersicum*) or potato (*Solanum tuberosum*) through feeding by adult Colorado potato beetles (*Leptinotarsa decemlineata*) or by simple mechanical damage induces the accumulation of defensive proteinase inhibitors in both damaged (local) and distal undamaged (systemic) leaves. This phenomenon was later referred to as systemic defense, i.e. certain factors are transported from the damaged leaves to the systemic leaves, enabling the plant to adapt to the mobile feeding behavior of insects (Vega-Munoz et al. 2020). Although the identities of herbivory/wounding-induced systemic signals remain elusive, studies have revealed that the signals are regulated by various factors, including jasmonic acid (JA) signaling (Li et al. 2002) and Ca^2+^ and electrical signaling (Mousavi et al. 2013, Nguyen et al. 2018).

A dodder parasite can parasitize two or more adjacent host plants simultaneously. In this manner, the vasculatures of different host plants are indirectly connected through dodder bridges, allowing signaling molecules to travel between hosts and influence their physiology and environmental interactions. Hettenhausen et al. (2017) used the dodder *Cuscuta australis* to connect two adjacent soybean (*Glycine max*) plants, forming dodder-connected plant clusters (DCPCs). After 24 h of *Spodoptera litura* feeding on only one of the connected soybean plants (local), hundreds of differentially regulated genes (DEGs) were detected in the other, non-damaged soybean plant. Consistently, the systemically connected but undamaged soybean plants also exhibited increased resistance to *S. litura* larvae. Genetic analysis indicated that the JA pathway in the local plant is required for either production or transmission of the systemic signals (Hettenhausen et al. 2017). Similarly, Fichman et al. (2024) found that in an Arabidopsis-dodder-Arabidopsis DCPC, wounding an Arabidopsis leaf triggered rapid reactive oxygen species (ROS) production in the systemically connected Arabidopsis plant within 10 min.

Dodder parasites have a broad host range, spanning the monocotyledonous *Allium* to various eudicots species. In a DCPC comprising dodder (*C. australis*), Arabidopsis (*Arabidopsis thaliana*), and tobacco (*Nicotiana tabacum*), which are from three different families, wounding-induced systemic signals can travel from Arabidopsis to undamaged tobacco plants and activate their defenses responses, suggesting that conservation of the herbivory/wounding-induced systemic signals in eudicots (Hettenhausen et al. 2017). Although dodder typically fails to parasitize monocots, with the exception of *Allium*, when a dodder has a compatible host, it can nevertheless also develop haustoria on adjacent incompatible hosts, and the haustoria can even connect with the vasculature of the incompatible hosts. For example, Lei et al. (2021) used dodder (*C. campestris*) to connect maize (*Zea mays*) with Arabidopsis, spring onion (*Allium ascalonicum*) with maize, and even sword fern (*Nephrolepis cordifolia*) with tobacco. It was found that wounding one of the host plants could still activate defenses in the other dodder-connected host, indicating that the herbivory/wounding-induced systemic defense signals may be conserved across euphyllophytes. Such DCPCs comprising dodder-connected host plants that are phylogenetically very distant from each other offer a valuable system for exploring the evolution of systemic signals beyond herbivory/wounding-induced signals.

Wounding/herbivory-induced systemic signaling also occurs between dodder and host plants. Aphid (*Myzus persicae*) feeding on *C. australis* for 1 d resulted in only about 170 DEGs in the dodder but more than 1000 DEGs in the host soybean plant, indicating the transfer of aphid feeding-induced systemic signals from dodder to the host plant. Importantly, following this transfer, the soybean plant exhibited increased resistance to subsequent attack by the chewing insect cotton leafworm (*Spodoptera litura*) as well as the soybean aphid (*Aphis glycines*) (Zhuang et al. 2018).

### Salt stress-induced systemic signaling

Salt stress severely threatens plant survival. Within individual plants, salt treatment rapidly triggers and propagates Ca^2+^ signaling between different parts of the root in a ROS-dependent manner (Choi et al. 2014, Evans et al. 2016). Salt stress-induced systemic signaling also occurs between dodder-connected host plants.

Li et al. (2020) examined whether treating one host plant in a DCPC could enhance the salt tolerance of the other host plant. In DCPCs, each comprising two cucumber (*Cucumis sativus*) plants connected by a dodder *C. campestris*, treating one cucumber plant with NaCl solutions for 3 d highly increased the salt tolerance of the systemic cucumber plant, when challenged by 15 d of salt stress (Li et al. 2020). This suggests that certain salt-induced systemic signals were induced in the cucumber plant, and these signals moved through dodder to the other cucumber plant, where salt tolerance-related responses were activated (Li et al. 2020).

Recently, Zheng et al. (2025) used genetically modified tobacco plants, in which the genes *RBOHD* (*respiratory burst oxidase homolog D*, encoding a ROS-producing enzyme), *CCD8* (*carotenoid cleavage dioxygenase 8*, encoding an enzyme for strigolactone biosynthesis), and *ABA2* (*xanthoxin dehydrogenase*, involved in ABA biosynthesis) were respectively knocked out or silenced, to analyze the functions of ROS, strigolactone, and the ABA pathway in salt-induced systemic signaling within a DCPC. Intriguingly, these three signal pathways all negatively regulate the salt stress-induced systemic signals between tobacco hosts and the strigolactone and ROS signaling likely converge on the ABA pathway to regulate the interplant systemic signals (Zheng et al. 2025).

### Nutrient signaling

Nitrogen (N) and phosphorus (P) are important macronutrients for plants, but their limited availability and non-uniform distribution in soil often pose challenges. To adapt to the limited soil N or P, plants have evolved sophisticated local and systemic signaling mechanisms to optimize the uptake, assimilation, and transport of these nutrients (Chien et al. 2018, Wang et al. 2018, Zhao et al. 2023). Remarkably, N and P stress-related systemic signals can be transmitted between host plants via dodder parasites, influencing nutrient uptake and plant association with rhizosphere microorganisms.

In a cucumber-dodder-cucumber DCPC, one cucumber plant was treated with N deficiency, and the transcriptomes of the root and leaf of the other N-replete cucumber plant were analyzed to examine whether N stress activates interplant systemic signaling between dodder-connected hosts (Zhang et al. 2021). Many DEGs were found in the leaves (up to 1024) and roots (up to 2136) of N-depleted cucumber plants; intriguingly, the dodder-connected N-replete cucumber plants exhibited even greater numbers of DEGs in both leaves (up to 1383) and roots (up to 3514). Additionally, both N-depleted and N-replete host plants exhibited changes in genome DNA methylation (Zhang et al. 2021). Moreover, in a DCPC comprising *C. campestris*-connected cucumber and soybean plants, after treating the soybean plant with N stress, increased N-uptake activity was detected in the cucumber plant. These findings indicate that N-deficiency signals transmitted via dodder enhanced the N-uptake capacity of systemic hosts, accompanied by substantial transcriptomic and epigenetic changes (Zhang et al. 2021).

Inorganic phosphate (Pi) is the major form of P that plants take up from soil. In part because of the low availability and mobility of Pi in soil, most land plants are able to form symbiosis with mycorrhizae. Mycorrhizal fungi receive photosynthesis-derived carbon from their associated plants but provide P, N, and water to the host plants (Shi et al. 2023). Tobacco plants, e.g. can associate with the arbuscular mycorrhizal fungus (AMF) *Rhizophagus irregularis*. Zhao et al. (2025) used *C. campestris* to connect two tobacco hosts, and found that P deficiency in one host plant increased the colonization efficiency of AMF in root of the other, P-replete, plant. Thus, similar to N stress, P deficiency also activates certain systemic signals in the P-depleted plant, which migrate to the P-replete plant and induce symbiosis between AMF and tobacco. Genetic analyses indicated that microRNA399s (miR399s), which are known to be mobile signals during plant adaptation to P stress (Chien et al. 2018, Chiang et al. 2023), and strigolactones in the Pi-depleted tobacco plant, play a negative role in regulating the systemic signals. Moreover, in the Pi-replete tobacco, which received the Pi systemic signals, the signaling pathway involving miR399s and strigolactones also suppresses the symbiosis between tobacco root and AMF (Zhao et al. 2025). Sequencing of miR399s indicated that they can move bidirectionally between tobacco and dodder, suggesting that miR399s may play a role in dodder-mediated interplant Pi signaling (Zhao et al. 2025).

### Light stress-induced systemic signaling

Sudden large increases in light intensity are a stress to plants, and trigger rapid local and systemic Ca^2+^ and ROS waves and activation of electrical signaling (Miller et al. 2009, Gilroy et al. 2016). Fichman et al. (2024) applied fluorescence dyes, which indicate ROS, Ca^2+^, and cell membrane depolarization, to the Arabidopsis-dodder *C. campestris*-Arabidopsis DCPC, and then exposed one Arabidopsis leaf to high light levels. Within 10 min, strong fluorescence was detected in both the treated and systemic Arabidopsis plants, indicating that high light-induced systemic signals rapidly migrated through the whole plant, and through the dodder bridge and into the other host plant, where they activated ROS and Ca^2+^ bursts and cell membrane depolarization (Fichman et al. 2024).

### Bidirectional interplant systemic signaling

During growth and development and during plant adaptation to an ever-changing environment, different parts of a plant must communicate using systemic signals, and it is conceivable that these communications are continuous and reciprocal, so that different parts of a plant can adjust physiology in a coordinated manner. Recent evidence from DCPCs also suggested bidirectional systemic signaling between dodder and host and between dodder-connected hosts.

Cotton leafworm (*Spodoptera litura*) feeding on dodder (*C. campestris*), which in turn is parasitizing tobacco, induces about 160 DEGs in dodder. However, if the JA pathway is silenced in the host tobacco, only 56 DEGs are detected in dodder (Qin et al. 2019). Thus, it seems that certain JA-dependent systemic signals from the host plant also regulate the response of dodder to feeding insects. Subsequently, two studies using elaborately designed controls demonstrated that dodder conveys bidirectional systemic signals between hosts (Li et al. 2020, Zhang et al. 2021). Li et al. (2020) set up three groups of DCPCs: a control group, in which both hosts were given a high salt medium (CS+), a second control group, in which both hosts were cultivated in normal medium (CS−), and a treatment group, in which one host was given high salt medium (TS+), while the other was given normal medium (TS−). Specific comparisons between the transcriptomes of TS+ and CS+ and between TS− and CS− allowed the examination of bilateral systemic signaling between TS+ and TS− plants. Up to 220 DEGs were found between TS+ and CS+, and more than 1000 DEGs were identified between TS− and CS− (Li et al. 2020). Similarly, bidirectional N systemic signals were found in the study by Zhang et al. (2021).

These data reveal not only that the stress-treated plant sends systemic signals to the neighboring dodder-connected host plant, but that the other host plant correspondingly sends signals back to the stressed plant. These studies on bidirectional systemic signaling-mediated interplant communications have important implications for our understanding of plant physiology: in response to stress or developmental cues, different parts of plants, e.g. roots and shoots, are likely to send specific systemic signals to each other to communicate and finally finely adjust their physiology.

## Interplant Transfer of Biomolecules between Dodder and Host Plants

Systemic signals are typically small and diffusible molecules transported through phloem. Importantly, evidence from next-generation sequencing and proteomic analyses has demonstrated that mRNAs, small RNAs (sRNAs), and proteins are also exchanged between hosts and parasitic plants as well as between hosts in DCPCs. Some of these mobile molecules confer physiological and ecological functions, impacting the interactions between these plants and the adaptation of the plants in parasitization systems to the environment.

### mRNAs

The mRNA of maize homeodomain transcription factor *KNOTTED1* (*KN1*) was the first mRNA found to move between cells (Lucas et al. 1995). Phloem sap sampling and grafting studies have subsequently shown that many mRNAs are transported over long distances in plants (Kehr et al. 2021). For example, Z. Zhang et al. (2016) detected more than 3000 graft-transmissible mRNAs translocated from cucumber (*Cucumis sativus*) scions to watermelon (*Citrullus lanatus*) rootstocks.

In parasitic systems, Roney et al. (2007) used RT-PCR and microarrays to detected a few mRNAs moving from pumpkin (*Cucurbita maxima*) into dodder (*Cuscuta pentagona*), and David-Schwartz et al. (2008) also identified four mobile mRNAs that moved from the tomato host into *C. pentagona*. RNA-seq analysis on the foreign mRNAs in hosts and dodder provided a genome-wide overview of interplant trafficking of mRNAs. The first RNA-seq study revealed that in an Arabidopsis-*C. pentagona* parasitization system, Arabidopsis stems and dodder stems contained about 0.6% and 1.1% foreign mRNAs, respectively, among the total mRNAs (Kim et al. 2014). However, many fewer mobile mRNAs were detected in the tomato-*C. pentagona* system (Kim et al. 2014), possibly due to incompatibility between dodder and tomato (Hegenauer et al. 2016, Hegenauer et al. 2020, Yang et al. 2025). Later, analyses on the transcriptome data of hosts and parasites in Arabidopsis-*C. reflexa* (Thieme et al. 2015, Liu et al. 2020), cucumber-*C. campestris* (Zhang et al. 2021), cucumber-*C. australis* (Song et al. 2022), and soybean-*C. australis* systems (Liu et al. 2020, Zhang et al. 2024) all similarly indicated that thousands of mRNAs are exchanged between hosts and dodder parasites, although the total abundance of foreign mRNAs is often less than 1%.

It is yet unclear what properties of an mRNA determine its mobility. W. Zhang et al. (2016) proposed that tRNA-related sequences in mRNAs with stem-bulge-stem-loop structures determine mRNA mobility. Consistently, *GUS-tRNA* fusion mRNA and GUS activity were detected in dodder (*C. campestris*) parasitizing the Arabidopsis expression *GUS-tRNA*, but not in dodder parasitizing Arabidopsis expressing *GUS* alone (Park et al. 2021). Moreover, Arabidopsis mRNAs with 5-methylcytosine (m^5^C) modifications are highly enriched in previously identified mobile mRNAs (Yang et al. 2019). To what degree the interplant-transferred mRNAs in host-parasitic plant systems are modified with m^5^C is an interesting field for future study.

Recent mathematical modeling has questioned the extent of mRNA mobility in grafting systems (Hoerbst et al. 2025, Paajanen et al. 2025). However, unlike grafting, which requires phylogenetically closely related species, plants in parasite–host systems are often very distantly related. This greatly facilitates identification of foreign/mobile and native mRNAs, although more rigorous bioinformatic analysis and controls (e.g. contamination controls) are still needed to reevaluate the landscape of mRNAs transported between hosts and parasites. Moreover, it would be valuable to study to what degree the inter host–parasite mobile mRNAs are translated into proteins and the functions of these translated proteins in the recipient plants.

### Small RNAs

sRNAs are 20 to 24-nt RNA molecules, and play important roles in plant growth and development as well as in the stress response (Zhan and Meyers 2023). There are three major pathways of sRNA biogenesis in plants: (i) 20–22 nt microRNAs (miRNAs) produced by dicer-like 1 (DCL1); (ii) 21 and 22 nt small interfering RNAs (siRNAs) respectively produced by DCL4 and DCL2; (iii) 24 nt heterochromatic siRNAs produced by DCL3 (Lee and Carroll 2018). sRNAs silence their target genes at the DNA and RNA levels by repressing the complementary sequences.

Previous studies have suggested that some miRNAs and siRNAs from transgenes with inverted-repeat sequences are transported over long distances, e.g. between leaves and between shoots and roots (Yan and Ham 2022). Shahid et al. (2018) detected many novel miRNAs with an uncommon length of 22 nt in the haustoria of *C. campestris* infesting Arabidopsis, with some of these miRNAs targeting Arabidopsis genes, including *TIR1*, *AFB2*, and *AFB3* (encoding auxin receptors), *BIK1* (encoding a membrane-localized kinase involved in both immunity and development), *SEOR1* (encodes an abundant phloem protein that reduces photosynthate loss from the phloem after injury), and *HSFB4* (a transcriptional repressor required for ground-tissue stem cell formations in root) (Shahid et al. 2018). Importantly, increased dodder biomass was detected in the *seor1* and *afb3-4* mutants, indicating that dodder uses specific miRNAs as virulence factors to target host development- and defense-related genes for parasitism. The authors also compared the miRNA profiles and the miRNA-target mRNAs when dodder was grown on Arabidopsis and on *Nicotiana benthamiana*, and found that the same dodder miRNAs target conserved host mRNAs (Shahid et al. 2018).

Johnson et al. (2019) further used four different dodder species, *C. campestris*, *C. pentagona*, *C. gronovii*, and *C. indecora* to infest Arabidopsis plants, and sRNA profiling indicated that all these dodder species use interplant sRNAs, probably mostly miRNAs produced in haustoria, to silence many host genes, including genes related to immunity, hormone pathways, and vascular functions. Interestingly, their analyses indicated that dodder evolved to produce haustorial sRNAs in superfamilies with a large nucleotide diversity which corresponds with target site variation primarily at synonymous sites. In this manner, dodder use sRNAs to facilitate its parasitization on various species.

### Proteins

More than 20 years ago, Haupt et al. (2001) detected GFP signals in *C. reflexa* parasitizing GFP-expressing tobacco (*Nicotiana tabacum*). Similarly, GFP signals were detected in *Phelipanche aegyptiaca*, whose hosts were tobacco plants expressing GFP (Aly et al. 2011). Another study, Jiang et al. (2013), found that dodder (*Cuscuta pentagona*) parasitizing transgenic soybean plants expressing the herbicide glufosinate-tolerant gene *phosphinothricin acetyl transferase* (*PAT*) became tolerant to glufosinate. There was no evidence of *PAT* mRNA transfer from the host, indicating transfer of PAT protein from host soybean to dodder.

Liu et al. (2020) used proteomics analysis to examine the translocation of proteins between host plants and dodder (*C. australis*). In Arabidopsis-dodder and soybean-dodder parasitization systems, hundreds to more than 1500 proteins were found to be mobile from host to dodder or vice versa. Furthermore, seeds from dodder and its host soybean were analyzed, and a few hundred foreign proteins were identified in these seeds (Liu et al. 2020). In a DCPC, in which a dodder parasite connected an Arabidopsis and a soybean plant, almost 800 and 1000 soybean and Arabidopsis proteins were identified in Arabidopsis and soybean leaves, respectively (Liu et al. 2020). Importantly, estimation of abundance of foreign proteins indicated that these interplant mobile proteins often quantitatively occupy a few percent of the total proteins (up to 11.17%), suggesting that proteins may be a major class of macromolecules transported between dodder and host plants. Comparison of the interplant mobile mRNAs and proteins indicated that most of these mobile proteins are not translated from the mobile mRNAs. Thus, most of the mobile proteins were directly transferred between plants and were not the result of translation of mobile mRNAs, a mechanism which was also found in a study using Arabidopsis homografts (Paultre et al. 2016).

Statistically, at least 10% of the interplant mobile proteins retained at least 50% of their abundance in their native plants, after they were translocated to the recipient plants (Liu et al. 2020). Liu et al. (2020) also used transgenic Arabidopsis or soybean plants expressing eGFP, eGFP-GUS fusion, luciferase, or allene oxidase synthase-FLAG fusion as the reporter plants. These proteins or their respective activity were then detected in the infested dodder plants. The implications of this study are far-reaching: large numbers of proteins in considerable quantities are transferred between hosts and dodder, and these proteins are likely to retain their biochemical activity. These transferred proteins may be family-, genus-, or species-specific, and they may be novel proteins to the recipient plants and thus affect the recipient plants’ physiology and ecology.

Flowering locus T (FT) protein regulates plant flowering (Turck et al. 2008). Under inductive conditions, the synthesis of FT in leaves is activated, and through phloem transport, FT reaches shoot apical meristems. In the apical meristems, FT physically interacts with the transcription factor flowering locus D (FD) to activate the transcription of flower organogenesis-related genes (Abe et al. 2005). Sixty years ago, Fratianne (1965) reported that dodder *C. campestris* flowered only when the host plants were grown under flowering-inductive photoperiod settings, although the underlying mechanisms remained unclear. Using different host species, which flower at different times or are sensitive to different photoperiods, and using genetically manipulated host plants whose flowering times were altered by overexpression or knocking out the *FT* gene, Shen et al. (2020) confirmed that the flowering times of dodder *C. australis* synchronized well with those of the hosts. Biochemical evidence suggests that host plant FT proteins, induced either under inductive photoperiod conditions or artificially using a chemically inducible promoter, can be translocated into dodder, where they interact with the *C. australis* FD and thus activate flowering (Shen et al. 2020). These findings reveal a novel aspect of dodder adaptive evolution: using the interplant-transported FT proteins from the hosts, dodder can synchronize its flowering time well with that of the host, allowing the parasite to achieve optimal fitness on different host species. The unique flowering mechanism of *C. australis* also well aligns with the finding that many flowering regulatory genes have been lost during the evolution of *C. australis* (Sun et al. 2018). Interestingly, a recent study into *C. campestris* flowering argued against the model of host FT-driven flowering in dodder (Mackelmann et al. 2024). Strikingly, in this study, bioinformatic analysis of the *C. australis* and *C. campestris* genome did not reveal obvious loss of flowering genes, and contrary to the findings of Fratianne (1965) or Shen et al. (2020), the flowering times of *C. campestris* were similar on WT and transgenic tobacco plants where the flowering times had been genetically manipulated to be shortened or delayed. It would be very interesting to reanalyze the genome data of *Cuscuta* species for flowering-related genes, especially using high-quality genome assemblies (e.g. Sun et al. (2018) for *C. australis* and PRJCA041563 deposited at the Beijing Institute of Genomics for *C. campestris* (Wu laboratory, unpublished)). More experiments are needed to further reveal the mechanisms underlying the regulation of flowering in dodders.

Given the critical role of proteins in plant physiology and the extent of protein transfer between hosts and dodder and between dodder-connected hosts, it is expected that many other interplant-transferred proteins playing important roles in host-dodder interactions will be found. It would also be interesting to investigate the transfer of proteins in other host–parasite systems, e.g. between hosts and *Orobanche* or *Phelipanche*.

## Perspective

Parasitic plants use various means to interact with their host plants, including systemic signals, proteins, mRNAs, and sRNAs. What are these systemic signals and how do they convey “information” and coordinate to regulate host or parasite physiology? Thus far, very little is known about these fascinating signaling molecules and their roles in interplant host–parasite interactions.

Host–parasitic plant interaction systems have become good models to study interplant transfer of macromolecules including mRNAs, sRNAs, and proteins. In addition to genetically modifying host plants, the hairy root transformation system for the hemiparasite *Phtheirospermum japonicum* (Orobanchaceae) is already available (Ishida et al. 2011), and recently the transformation protocol for *C. campestris* has been reported (Adhikari et al. 2024). These genetic tools and the increasing numbers of high-quality reference genomes of parasitic plants will provide exciting opportunities for researching the functions of these interplant-transferred macromolecules in host plant-parasitic plant interactions.

It is worth mentioning that most hemiparasitic plants form xylem connections with the host plants, but not phloem connections. In host plant-hemiparasite systems (e.g. Arabidopsis-*P. japonicum*), are the various molecules (systemic signals, RNAs, and proteins) transported between host and parasite through the xylem tissue as well, and if so, how are they able to impact the recipient’s physiology? This is a fascinating question that remains to be explored.

## Data Availability

No datasets were generated or analyzed in this study.
